# Effects of Association of Barley Plants with Hydrocarbon-Degrading Bacteria on the Content of Soluble Organic Compounds in Clean and Oil-Contaminated Sand

**DOI:** 10.3390/plants10050975

**Published:** 2021-05-13

**Authors:** Sergey Chetverikov, Lidiya Vysotskaya, Elena Kuzina, Tatiana Arkhipova, Margarita Bakaeva, Gulnaz Rafikova, Tatiana Korshunova, Darya Chetverikova, Gaisar Hkudaygulov, Guzel Kudoyarova

**Affiliations:** Ufa Institute of Biology, Ufa Federal Research Centre, Russian Academy of Sciences, 450054 Ufa, Russia; che-kov@mail.ru (S.C.); vysotskaya@anrb.ru (L.V.); misshalen@mail.ru (E.K.); tnarkhipova@mail.ru (T.A.); margo22@yandex.ru (M.B.); rgf07@mail.ru (G.R.); korshunovaty@mail.ru (T.K.); belka-strelka8031@yandex.ru (D.C.); bio.logos@yandex.ru (G.H.)

**Keywords:** plant-bacteria association, petroleum contamination, phytoremediation, bacterial oil degradation, root exudates, phytohormones, *Enterobacter*, *Pseudomonas*, *Hordeum vulgare* L.

## Abstract

Plant-bacteria consortia are more effective in bioremediation of petroleum contaminated soil than when either organism is used individually. The reason for this is that plant root exudates promote growth and activity of oil degrading bacteria. However, insufficient attention has been paid to the ability of bacteria to influence root exudation. Therefore, the influence of barley plants and/or bacterial inoculation (*Pseudomonas hunanensis* IB C7 and *Enterobacter* sp. UOM 3) on the content of organic acids, sugars and plant hormones in the eluate from clean and oil-polluted sand was studied separately or in combination. These strains are capable of oxidizing hydrocarbons and synthesizing auxins. Concentrations of organic acids and sugars were determined using capillary electrophoresis, and hormones by enzyme-linked immunosorbent assays. In the absence of plants, no sugars were detected in the sand, confirming that root exudates are their main source. Introducing bacteria into the sand increased total contents of organic compounds both in the presence and absence of oil. This increase could be related to the increase in auxin amounts in the sand eluate, as well as in plants. The results indicate that bacteria are able to increase the level of root exudation. Since auxins can promote root exudation, bacterial production of this hormone is likely responsible for increased concentrations of soluble organic compounds in the sand. Bacterial mediation of root exudation by affecting plant hormonal status should be considered when choosing microorganisms for phytoremediation.

## 1. Introduction

Deposition of soluble organic compounds into the soil in the form of root exudates plays an important role in stimulating the growth and reproduction of bacteria in the rhizosphere, since secretion of nutrients provides nitrogen and carbon sources to soil microorganisms [1,2,3]. Root exudation was shown to be increased by stress, contributing to bacterial growth under unfavorable environments [4,5]. In particular, soluble organic compounds produced by plants and exuded by their roots into the rhizosphere provide microorganisms with a source of energy for the oxidation of toxic oil hydrocarbons, thereby increasing the efficiency of oil biodegradation. [6,7,8]. At the same time, the effectiveness of plant-bacteria associations during the process of phytoremediation is determined by their interaction. Plants not only provide bacteria with root exudates, but bacteria can also influence the growth of plant roots [9] thereby determining the process of exudation [6,10]. Hormones produced by bacteria play an important role in this regard. It has been shown that auxins [11] and cytokinins [12] increase root exudation. Although exudation was previously assumed to be a passive process via diffusion [13,14] depending only on the size of the root system [15], nowadays researchers are inclined to believe that plants are able to control the level of root exudation by changing the activity of carrier proteins of sugars and other organic compounds [16]. In experiments with *Arabidopsis* plants, a decrease in exudation was shown in the mutant with a defective ABC transporter (ATP-binding cassette transporters) [17]. Since the plant hormone auxin can activate ATPases [18], indole-3-acetic acid (IAA, a plant auxin) produced by bacteria may be involved in the regulation of ATPase dependent root exudation. However, when soil was contaminated with oil, the relationship between auxin production and root exudation has not yet been studied. Plant growth was shown to be influenced by some bacterial strains from genera *Pseudomonas* and *Enterobacter*, which are capable of oxidising oil hydrocarbons and producing auxins [19,20,21]. Their growth promoting effect was not only due to a decrease in the toxicity of oil components, but also to the immediate effect of bacteria on plant growth brought about by their ability to produce plant hormones [20,22]. However, effects of these bacterial strains on root exudation have not been studied under normal conditions and oil pollution.

This work aims to reveal the effects of *P. hunanensis* IB C7 and *Enterobacter* sp. UOM 3 on the accumulation of organic acids and sugars in the substrate under conditions of oil pollution and the presence of barley seedlings. These strains were characterized by a relatively high activity in oxidation of oil components and ability to produce IAA [20]. Taking into account the presumptive role of hormones in the regulation of root exudation, the concentration of hormones in the substrate solution and in plants was analyzed.

## 2. Results

### 2.1. Bacterial Abundance in the Sand

Throughout the experiment, in the treatment without bacterial inoculation, bacterial number in the sand was low. The number of heterotrophic and hydrocarbon-oxidizing bacteria in the sand sharply increased upon inoculation with the tested bacterial strains. These results suggest that the introduced strains made the main contribution to the total number of microorganisms (Table 1). At the end of experiments, treatments without oil had a high abundance of both *P. hunanensis* IB C7 and *Enterobacter* sp. UOM 3. (Table). In the presence of oil, the abundance of both oil degrading bacteria *P. hunanensis* IB C7 and Enterobacter sp. UOM 3 increased 5 and 10 times on the 14th day, respectively.

### 2.2. Content of Organic Acids in the Sand Eluate

**Figure 1:**
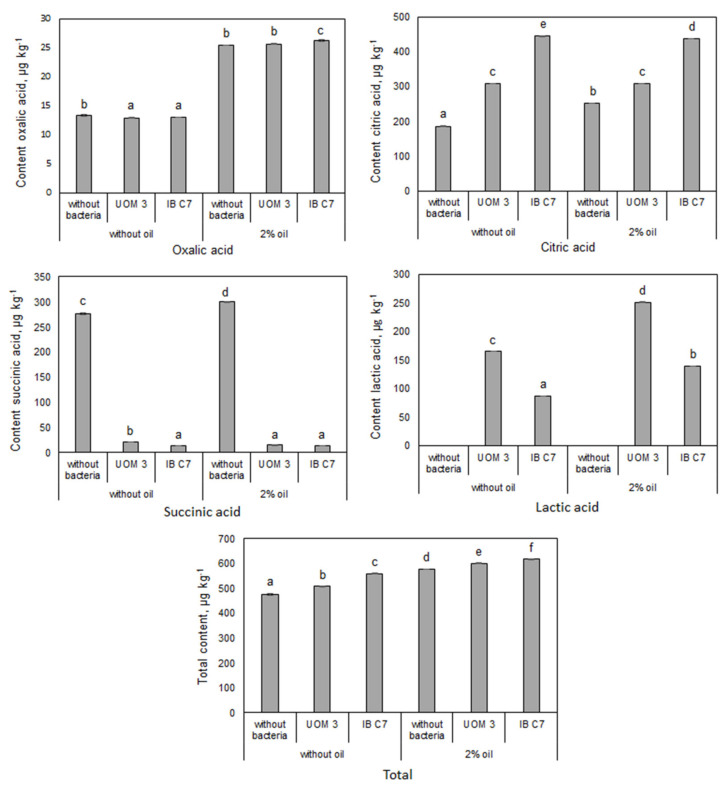
Content of organic acids in pots planted with barley, μg kg^−1^ of sand. UOM 3 and IB C7—treatments with introduction of *Enterobacter* sp. UOM 3 and *P. hunanensis* IB C7, respectively. Statistically different means for each organic acid (n = 9) are marked with different letters (*p* ≤ 0.05).

In eluates from unplanted sand with introduced bacteria, only malic acid was detected at a low concentration (about 2 μg kg^−1^), while the presence of other organic acids and sugars was not detected. The eluate from planted soil was characterized by even lower content of malic acid (less than 1.25 μg kg^−1^) (data not shown in the table). Without bacterial inoculation, succinic acid dominated in the planted sand, comprising about 50% of the total of all organic acids, while lactic acid was absent (Figure 1).

Introducing bacteria greatly (more than ten times) decreased succinic acid content in the sand, increased citric acid content and led to the appearance of lactic acid, which was not found in planted sand not inoculated with bacteria (Figure 1). Lactic acid contents were higher with *Enterobacter* sp. UOM 3 rather than *P. hunanensis* IB C7 inoculation. In the absence of oil, the amount of citric acid increased under the influence of *Enterobacter* sp. UOM 3 and *P. hunanensis* IB C7 by 1.7 and 2.4 times, respectively, and its percentage in the total content of identified organic acids increased to 60 and 80%, respectively.

Under oil contamination, the total amount of organic acids detected in the sand increased by about 20%, while the citric acid content increased by 35% in sand without introduced bacteria. The content of oxalic acid was increased almost doubled by the presence of oil, but since its content in the sand was an order of magnitude lower than that of citric acid; its contribution to the pool of organic acids was small. Introducing bacteria into petroleum-contaminated sand was accompanied by an additional increase in the content of citric acid by 22% in the sand with *Enterobacter* sp. UOM 3 and 74% in the case of *P. hunanensis* IB C7.

### 2.3. Content of Monosaccharides in the Sand Eluate

**Figure 2:**
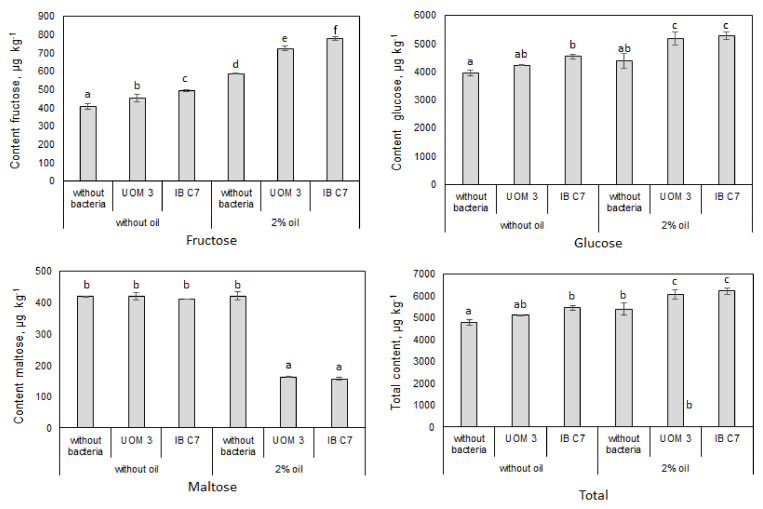
Content of sugars in pots planted with barley, μg kg^−1^ of sand. UOM 3 and IB C7—treatments with introduction of *Enterobacter* sp. UOM 3 and *P. hunanensis* IB C7, respectively. Statistically different means for each sugar (n = 9) are marked with different letters (*p* ≤ 0.05)

The total content of monosaccharides in the sand eluate was an order of magnitude higher than that of organic acids (Figure 2). The level of fructose increased under the influence of both bacteria and the presence of oil, while maltose, on the contrary, decreased 2.6–2.7 times if bacteria and oil were present simultaneously. The increase in the fructose content under the influence of the *P. hunanensis* IB C7 strain was 32% with oil contamination, and 21% without. In the absence of oil, *P. hunanensis* IB C7 inoculation significantly increased glucose content, while in the presence of oil its content was increased by both strains. In general, treatment with *P. hunanensis* IB C7 was more effective than *Enterobacter* sp. UOM 3.

### 2.4. Content of Hormones in the Sand Eluate and in Plants

**Figure 3:**
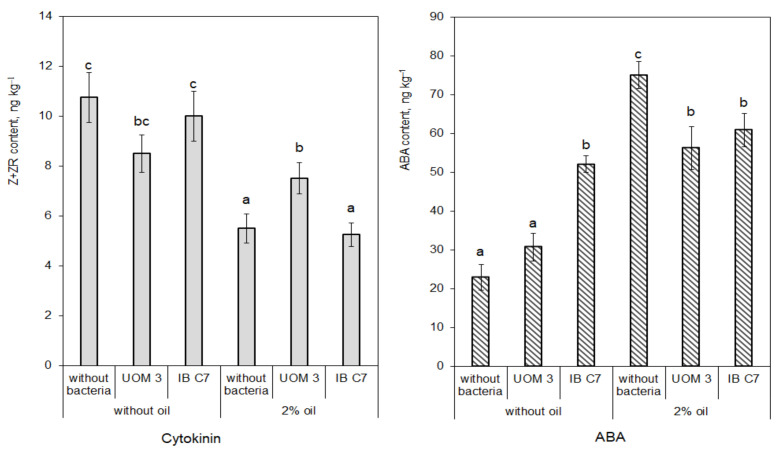
Cytokinin (Z+ZR) and ABA content pots planted with barley, ng kg^−1^ of sand. UOM 3 and IB C7—treatments with introduction of *Enterobacter* sp. UOM 3 and *P. hunanensis* IB C7, respectively. Statistically different means for each hormone (n = 9) are marked with different letters (*p* ≤ 0.05).

Cytokinins were not detected in the eluate from the unplanted sand, while ABA content was at the limit of the method’s sensitivity (data for the content of these hormones in unplanted sand are not shown). In the presence of plants, contents of these hormones remained low (no more than 12.5 μg kg^−1^ for cytokinins and 75 μg kg^−1^ for ABA) (Figure 3). The amount of cytokinins decreased, while ABA increased under the influence of oil pollution (Figure 3). In planted oil-polluted sand, ABA content was three times higher than without oil, when bacteria were absent.

**Figure 4:**
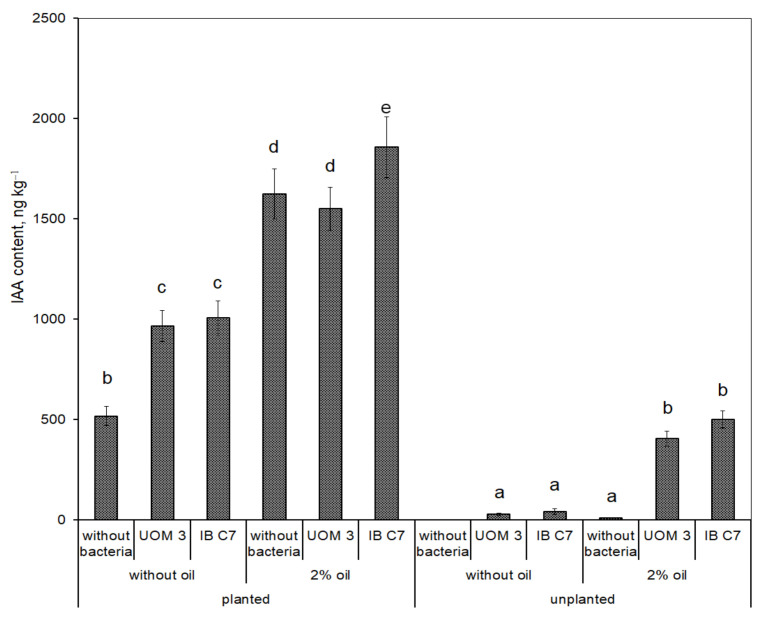
Indole-3-acetic acid content in pots planted and unplanted with barley, ng kg^−1^ of sand. UOM 3 and IB C7—treatments with introduction of *Enterobacter* sp. UOM 3 and *P. hunanensis* IB C7, respectively. Statistically different means (n = 9) are marked with different letters (*p* ≤ 0.05).

The IAA content in the eluate from the planted sand was about 500 ng kg^−1^, which is an order of magnitude higher than that of ABA and cytokinins (Figure 4). It increased in the presence of oil to 1625 ng kg^−1^, but bacterial treatment influenced IAA concentrations only in the absence of oil, when IAA increased two times compared with the treatment without bacteria. A similar increase was detected when either strain was used. In clean unplanted sand with introduced bacteria, IAA content was 20 times lower than in the presence of plants. Oil pollution increased the amount of IAA in unplanted, but inoculated sand up to 500 ng kg^−1^.

**Figure 5:**
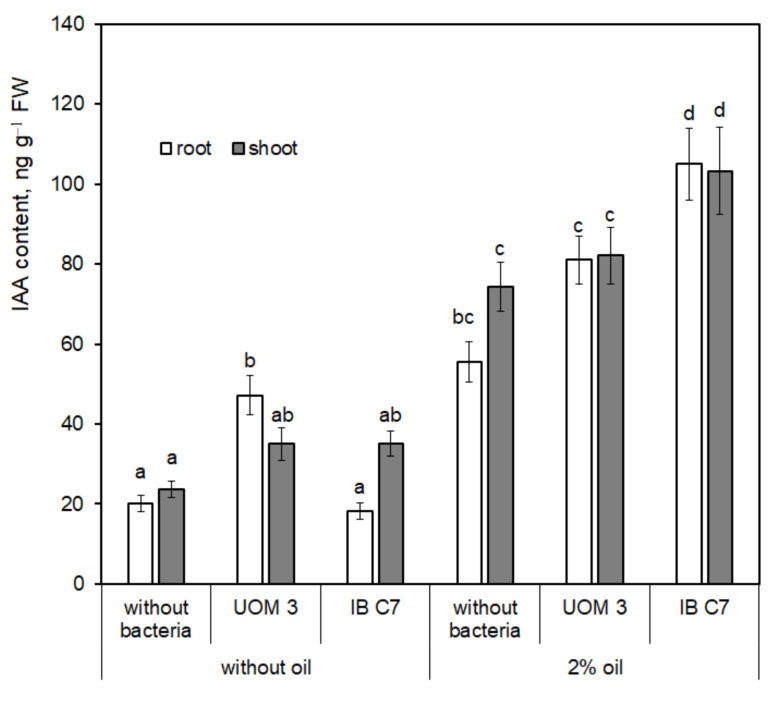
Indole-3-acetic acid content in shoots and roots of barley plants (ng g^−1^ fresh weight). UOM 3 and IB C7—treatments with introduction of *Enterobacter* sp. UOM 3 and *P. hunanensis* IB C7, respectively. Statistically different means (n = 9) are marked with different letters (*p* ≤ 0.05).

The content of auxins in plants mainly increased under the influence of either oil or bacterial treatment (Figure 5). When grown in petroleum contaminated sand, root IAA concentration of plants inoculated with *P. hunanensis* IB C7 was almost 2 times higher than in plants grown without bacteria.

### 2.5. Effect of Oil on Shoots and Roots Biomass

**Figure 6:**
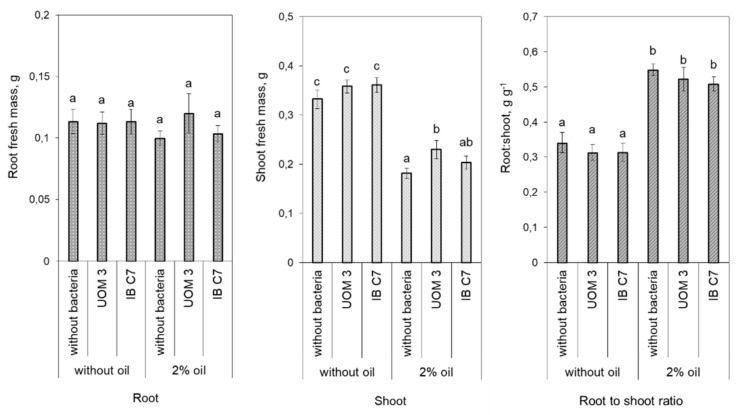
Root and shoot mass and root:shoot ratio in barley plants. UOM 3 and IB C7—treatments with introduction of *Enterobacter* sp. UOM 3 and *P. hunanensis* IB C7, respectively. Statistically different means for each characteristic (n = 18) are marked with different letters (*p* ≤ 0.05).

The presence of oil greatly decreased shoot mass compared to plants that grew in clean sand, but did not affect the mass of the roots, resulting in an increased root-to shoot ratio (Figure 6).

## 3. Discussion

Bacterial abundance in the sand was mostly due to the introduced strains. By the end of the experiment, the number of introduced microorganisms remained at a high level in the treatment without oil, indicating their high survival rate in the rhizosphere of barley. As expected, the introducing oil into the sand stimulated the growth of microorganisms that degrade hydrocarbons, in accordance with the literature [23,24].

The absence of sugars and most organic acids in unplanted sand agrees with reports that plants serve as the primary source of soluble organic compounds in the rhizosphere [25], confirming the reliability of the measurements. At the same time, introducing bacteria into the rhizosphere affected the composition of organic compounds: the content of succinic acid in the eluate from the sand sharply decreased, while that of citric acid increased, and lactic acid appeared. Bacterial introduction also increased fructose and glucose concentrations, although to a lesser extent than that of organic acids. In the presence of oil, concentrations of organic compounds also increased, consistent with literature data of an increase in root exudation under stress conditions [4,5]. The increase in the content of organic acids under the influence of oil pollution was more noticeable when plants were bacterized.

Bacterial metabolism (synthesis of some compounds and catabolism of others) may influence compositional changes in the organic compounds. Petroleum hydrocarbons can serve as the source for the synthesis of organic compounds. In this way, acylCoA produced as a result of β-oxidation of aliphatic chains of petroleum hydrocarbons enters the Krebs cycle and can contribute to the accumulation of citric acid [26]. Greater increase in citric acid in the pool of organic acids in the eluate from sand was detected against the background of introducing the *Pseudomonas* strain. Interestingly, bacteria of this genus produce chemotactic peptides that react with citric acid, thereby promoting bacterial attraction to the root surface and its colonization [6]. In the presence of oil and bacteria, maltose concentration in the eluate sharply decreased, which may indicate active microbial utilization of this sugar under stressful conditions to support bacterial growth. Introducing bacteria increased glucose and fructose concentrations, suggesting that bacterial promotion of root exudation of these compounds outweighed their consumption by bacteria. According to some reports, adding glucose to the incubation medium increases bacterial ability to degrade oil components [27]. Although in the present experiments the increase in glucose concentration under the influence of oil pollution was less than that of fructose, it is possible that the lesser glucose increment reflects its more active metabolism by bacteria than that of fructose.

The changes in the composition of organic constituents in the eluate from sand may result not only from the metabolic activity of bacteria, but also due to changes in the quantity and quality of root exudates secreted by plants. Thus concentration of root exudates increased under the influence of oil in the absence of introduced bacteria (Figure 1 and Figure 2).

Although the role of root exudates in the regulation of bacterial abundance and activity is well known [1,2,3], less attention has been paid to the reverse effect of bacteria on root exudation, and there is little information on how bacteria can alter root exudation [4,13]. The results obtained in the present experiments confirm that bacteria can increase root exudation both in the presence and absence of oil in the sand. It was important to relate these effects to the changes in hormone content in the sand and plants. While cytokinins produced by *Bacillus subtilis* stimulate exudation of amino acids [12], against the background of oil pollution, no change in cytokinin levels were detected in barley plants [21], and concentrations of cytokinins in the eluate from the sand were low (Figure 3). Therefore, the results of IAA analysis were of greater interest.

Auxins can be produced by both plants and bacteria and can increase root exudation [11]. Interestingly, the increase in the concentration of IAA either in the eluate from sand or in the plants themselves was detected in oil-contaminated sand, which was accompanied by and may indeed cause an increase in concentration of soluble organic compounds in the sand. Auxins’ effects on root exudation may be due to their influence on root growth [28]. Root mass was increased by oil pollution when barley plants were grown in soil [21]. However, in the present experiments carried out in a sand culture, root mass did not increase in stressed plants (Figure 6). Therefore, the increase in the concentration of organic compounds detected in the present experiments in the eluate from the sand reflects changes in root exudation independent of root mass. Involvement of putative plant transporters in root exudation of sugars and organic acids has been recently reviewed [29]. Auxin can activate membrane ATPase [18,30], enabling the transport of substances against their concentration gradient [16]. Sugars are transported in symport with hydrogen ions accumulated in the apoplast due to plasma membrane H^+^−ATPase activity [31]. Several researchers argue that the root exudation of sugars is passive [16]. However, an increase in auxin concentration promotes the outflow of sugars to the roots [32], which should affect root exudation. Furthermore, plants are able to control root exudation by regulating activity of transporters, including ATP-binding cassette transporters [2,17].

Both bacterial strains were able to synthesize auxins [20]. However, IAA levels in the eluate from sand with bacteria was low in the absence of plants (Figure 5). Nevertheless exudation of some endogenous plant signals may enhance auxin production by bacteria growing in the vicinity of the roots [33]. Therefore, it is not surprising that IAA concentrations in the eluate from the sand increased as a result of bacterization (in the absence of oil) (Figure 4), which could contribute to increased root exudation resulting in higher concentration of organic compounds in the sand eluate (Figure 1 and Figure 2). However, no bacteria-induced increase in IAA concentration was detected in the eluate from petroleum contaminated sand. At the same time, plant IAA concentrations increased both under the influence of oil and bacterization (Figure 5). Obviously, in the non-bacterial treatment, the petroleum induced increase in IAA content both in the eluate and in plants was a consequence of plant auxin synthesis. However, the additional bacteria-induced auxin increment *in planta* may be explained by the ability of bacteria to produce auxin and its uptake by plants. Although bacterization did not increase IAA level in the eluate from petroleum contaminated sand, bacterial activation of plant auxin uptake [34] may enhance plant IAA levels.

## 4. Materials and Methods

### 4.1. Plant Growth Conditions and Treatments

Barley plants (*Hordeum vulgare* L., cv. Chelyabinskiy 99) were used to study the effects of plant-bacteria association on soluble organic compounds in a sand growing substrate in the presence and absence of petroleum. In preliminary experiments comprising 6 plant species, barley was chosen as the most petroleum-resistant and the most responsive to bacterial inoculation in terms of growth promoting effect [20]. Six barley seedlings, whose coleoptile had a length of 0.5–1 cm, were planted in each 500 mL vessel with clean or oil-contaminated sand. The sand had already been sterilized by calcinations to prevent introduction of undesirable bacteria and saturated with Hoagland-Arnon solution containing 0.5 mM KNO_3_, 0.5 mM Ca(NO_3_)_2_, 0.1 mM KH_2_PO_4_, 0.1 mM MgSO_4_ and essential microelements. Germinated seedlings were sprinkled on top of the sand with an appropriate substrate and placed on a light platform equipped with red (650 nm), blue (470 nm) and white LEDs (in a ratio of 5:1:1), providing illumination of 240 μmol m^−2^ s^−1^ PAR. Throughout the experiment, the plants grew at a temperature of 22–26 °C and a 14-h photoperiod. Petroleum pollution of the soil was achieved by adding oil (Ural brand) up to 2% by weight to dry sand after which the mixture was uniformly mixed for 20 min. Water content was maintained at 60–90% of sand water-holding capacity by daily replenishing evapotranspiration losses. Before planting in the soil containing oil or without it, the barley seedlings were soaked for 20 min in a liquid culture of *P. hunanensis* IB C7 and *Enterobacter* sp. UOM 3 diluted with water to 10^6^ CFU g^−1^. Bacterial liquid cultures were additionally added into the sand to reach concentration of 2–3 × 10^6^ CFU g^−1^. Two weeks after the start of experiment, shoot and root biomass of barley plants were measured.

### 4.2. Bacterial Strains and Culture Media

Bacterial strains of *P. hunanensis* IB C7 and *Enterobacter* sp. UOM 3 have been previously selected from the collection of Ufa Institute of Biology, Ufa Federal Research Centre, Russian Academy of Sciences (UIB UFRC RAS) as being capable of oxidizing oil products and accumulating indole-3-acetic acid (IAA) in vitro [20,35]. Bacteria were cultured for 72 h in meat-peptone broth (g L^−1^): peptone—5, NaCl—5, at a temperature of 28 °C; aeration of the medium was provided by rotating flasks (160 rpm) in an orbital shaker-incubator ES-20/60 (SIA BIOSAN, Riga, Latvia). The number of cells in the culture was measured by applying serial dilutions to the nutrient agar (g L^−1^): peptone—10, yeast extract—3, NaCl—5, glucose—1, agar-agar—15 and then counting the number of colony forming units (CFU).

In order to estimate the microbial counts in sand, a serial dilution of sand eluate was used. The number of heterotrophic microorganisms was measured by application to the nutrient agar (see above). For measuring the number of petroleum degrading bacteria, Raymond agar (g L^−1^): NH_4_NO_3_ − 2.0, MgSO_4_ × 7H_2_O − 0.2, KH_2_PO_4_ − 2.0, Na_2_HPO_4_ − 3, CaCl_2_ × 6H_2_O − 0.01, Na_2_CO_3_ − 0.1, agar-agar − 15, pH − 7.0, supplemented with 0.1 g diesel fuel as the only source of carbon, smeared on the agar surface of each plate was used. The incubation period at 28 °C was five days on the Raymond agar plate and three days on nutrient agar. The average number of colonies was calculated in ten agar plates.

### 4.3. Analysis of the Content of Organic Acids and Monosaccharides in the Sand Eluate

**Figure 7:**
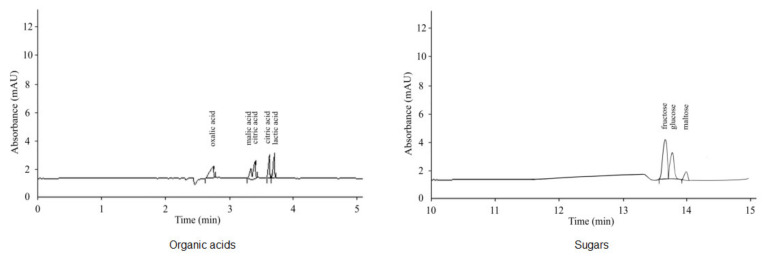
Electrophoregrams of a standard mixtures of organic acids (40 mg L^−1^ each) and sugars (500 mg L^−1^ each).

Two weeks after shoot emergence, contents of organic acids and sugars in eluates from sand were evaluated. To collect sand eluates, 50 mL of distilled water was poured in portions onto the surface of the sand and the eluate flowing out of the vessel was collected, in which the contents of organic compounds were determined in 100 µL per sample (weight/vessel). The analysis of sugars and organic acids in soil solutions and plant extracts was carried out using the method of capillary electrophoresis [36,37] and this method was used in the present research. The instructions of the manufacturer of a Kapel-104 RT system (Lumex, St. Petersburg, Russia) with an ultraviolet detector, wavelength of 254 nm, quartz capillary with an effective length of 0.5 m and an inner diameter of 75 × 10^−6^ m were followed. Benzimidazole was used as electrolyte for the analysis of organic acids, while for the carbohydrates an electrolyte containing 0.5% potassium sorbate, 0.62% cetyltrimethylammonium bromide and 0.02% potassium hydroxide was used. The samples were inserted hydrodynamically with a pressure of 30 mbar for 5 s, in the case of sugars and 150 mbar for 1 s in the case of organic acids. The recommended negative voltage is 16 kV (for sugars) and 20 kV (for organic acids). The results were processed using the software “Multichrom for Windows, version 1.5” (LLC “Ampersand”, Moscow, Russia) according to the peaks obtained on electrophoregrams. Before analysis, samples containing plant root exudates were filtered through membrane filters with pores of 0.22 μm. Figure 7 shows the separation of the analyzed components.

### 4.4. Hormone Measurement

Two weeks after shoot emergence, the concentrations of IAA in shoots and roots of barley and in eluates from sand were evaluated. Five plants were pooled, then extracted with 80% ethanol for 16 h. After evaporation of the alcohol, IAA was partitioned from the aqueous residue into diethyl ether as decribed [31,38]. Indole-3-acetic acid and abscisic acid were partitioned from 5 mL of the sand eluate in the same way. Cytokinins (zeatin and its riboside) from sand eluate were purified as described [12]. Concentrations of hormones were measured by ELISA using the appropriate antibodies [39,40,41].

### 4.5. Statistical Analysis

The data were processed using the Statistica version 10 software (Statsoft, Moscow, Russia). In figures and tables, data are presented as mean ± standard error. The significance of differences was assessed by ANOVA followed by Duncan’s test (*p* ≤ 0.05).

## 5. Conclusions

Barley root exudation increased the concentrations of organic compounds in the sand and supplied the introduced bacteria with a substrate to allow growth. Addition of petroleum to the sand increased root exudation in parallel with an increase in abundance of introduced hydrocarbon degrading bacteria (*Pseudomonas hunanensis* IB C7 and *Enterobacter* sp. UOM 3) which should increase the efficiency of detoxification of petroleum hydrocarbons. The presence of bacteria increased concentration of organic compounds in the sand both in the absence of oil and in its presence suggesting that bacteria were capable of stimulating root exudation, thereby increasing substrate availability for their own growth. Bacteria increased concentrations of organic compounds in the sand solution, which was accompanied by and might be due to an increase in concentration of IAA detected both in the sand and barley plants. Although IAA likely exerted its effect by activating ATPases involved in the function of the ABC transporter, verifying this assumption should be a future research goal.

## Figures and Tables

**Table 1:** The number of bacteria in the sand, CFU g^−1^.

Group of Microorganisms		Treatments		
		Without Bacteria	*Enterobacter* sp. UOM 3	*P. hunanensis* IB C7
Without Oil				
Heterotrophic	1 day	0	(2.23 ± 0.13) × 10^6^	(1.89 ± 0.13) × 10^6^
	14 days	5.98 ± 0.22	(1.28 ± 0.09) × 10^5^	(2.51 ± 0.10) × 10^5^
Hydrocarbon-oxidizing	1 day	0	(2.12 ± 0.11) × 10^6^	(1.90 ± 0.12) × 10^6^
	14 days	0	(0.54 ± 0.12) × 10^5^	(1.42 ± 0.10) × 10^5^
2% oil				
Heterotrophic	1 day	0	(2.49 ± 0.11) × 10^6^	(2.92 ± 0.09) × 10^6^
	14 days	9.11 ± 0.19	(4.14 ± 0.08) × 10^7^	(1.56 ± 0.12) × 10^7^
Hydrocarbon-oxidizing	1 day	0	(2.52 ± 0.08) × 10^6^	(2.79 ± 0.13) × 10^6^
	14 days	5.13 ± 0.21	(3.08 ± 0.12) × 10^7^	(1.33 ± 0.09) × 10^7^

## Data Availability

The data presented in this study are available in the article.
